# Understanding Antiferromagnetic Coupling in Lead-Free Halide Double Perovskite Semiconductors

**DOI:** 10.1021/acs.jpcc.3c08129

**Published:** 2024-03-14

**Authors:** Kunpot Mopoung, Weihua Ning, Muyi Zhang, Fuxiang Ji, Kingshuk Mukhuti, Hans Engelkamp, Peter C. M. Christianen, Utkarsh Singh, Johan Klarbring, Sergei I. Simak, Igor A. Abrikosov, Feng Gao, Irina A. Buyanova, Weimin M. Chen, Yuttapoom Puttisong

**Affiliations:** † Department of Physics (IFM), Linköping University, Linköping 583 30, Sweden; ‡ Institute of Functional Nano & Soft Materials (FUNSOM), Soochow University, Suzhou 215123, P.R. China; § High Field Magnet Laboratory (HFML - EMFL), Radboud University, Toernooiveld 7, Nijmegen 6525 ED, The Netherlands; ∥ Department of Physics and Astronomy, Uppsala University, Uppsala SE-75120, Sweden

## Abstract

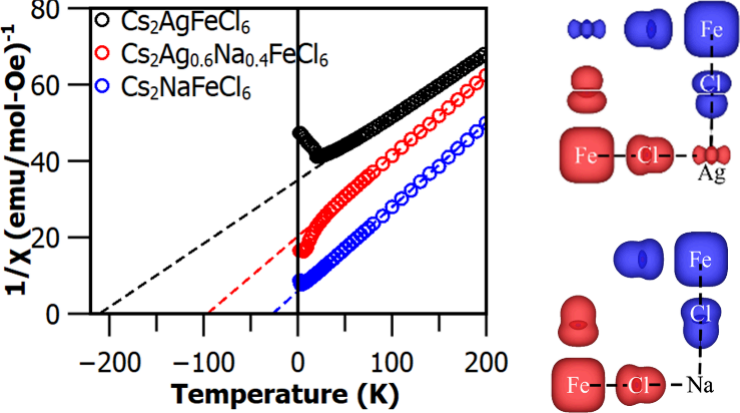

Solution-processable semiconductors with antiferromagnetic (AFM) order are attractive for future spintronics and information storage technology. Halide perovskites containing magnetic ions have emerged as multifunctional materials, demonstrating a cross-link between structural, optical, electrical, and magnetic properties. However, stable optoelectronic halide perovskites that are antiferromagnetic remain sparse, and the critical design rules to optimize magnetic coupling still must be developed. Here, we combine the complementary magnetometry and electron-spin-resonance experiments, together with first-principles calculations to study the antiferromagnetic coupling in stable Cs_2_(Ag:Na)FeCl_6_ bulk semiconductor alloys grown by the hydrothermal method. We show the importance of nonmagnetic monovalence ions at the B^I^ site (Na/Ag) in facilitating the superexchange interaction via orbital hybridization, offering the tunability of the Curie–Weiss parameters between −27 and −210 K, with a potential to promote magnetic frustration via alloying the nonmagnetic B^I^ site (Ag:Na ratio). Combining our experimental evidence with first-principles calculations, we draw a cohesive picture of the material design for B-site-ordered antiferromagnetic halide double perovskites.

## Introduction

Metal-halide perovskite (MHP) semiconductors have recently emerged as the dominant portfolio for high-performance solution-processable optoelectronics.^1^ Current state-of-the-art group IV-halide perovskites (with nonmagnetic Pb^2+^, Sn^2+^, and Ge^2+^) have already set the hallmark in high-performance solar cells,^2,3^ light-emitting diodes, lasers,^4,5^ and photodetectors.^6,7^ Recently, there has been an increasing interest in magnetic properties of halide double perovskites, with a grand scheme aiming at multifunctionalized magnetic semiconductors.^8−10^ Along this line, antiferromagnetic (AFM) order has increasingly become a sought-after property. This emerging research interest arises from the recent revisiting of applications of antiferromagnets in spintronics, which could complement or even surpass applications of ferromagnetic materials.^11−13^ During the past decade, we have witnessed a rise in the research interests in AFM semiconductors and AFM hybrid heterointerfaces.^14^ Their attractive features include a strong coupling of AFM magnons to both phonons and excitons,^15^ THz manipulation of spin,^12,16,17^ and an enhanced optical nonlinear effect.^18,19^ In addition, robust protection from stray magnetic fields due to the absence of net magnetization in the AFM phase provides a means to suppress electron spin dephasing that would benefit both spintronic and quantum computation applications.

A lead-halide perovskite is typically nonmagnetic, but the structural and chemical diversity of the MHPs allow the incorporation of magnetic ions that lead to magnetic response^20^ and the modification of the optical properties of nonmagnetic MHPs.^21,22^ More importantly, a structural derivative such as cesium halide double perovskites with a general formula of A_2_B^I^B^III^X_6_, where A is Cs^1+^ cations, B^I^ are monovalence metal cations (+1), B^III^ are trivalence metal cations (+3), and X are halide anions, e.g., Cl^1–^, Br^1–^, I^1–^, allows the search for new magnetic alloys by incorporating transition/rare-earth metal ions at the B^III^ site. Very recently, nontoxic and stable Cs_2_(Ag:Na)FeCl_6_ semiconductor alloys have been synthesized,^23^ demonstrating a rich functionality. The absorption edge of Cs_2_(Ag:Na)FeCl_6_ can be tuned from the visible to near-infrared spectral range by varying the Ag/Na ratio, which is attractive for solar cell applications.^23,24^ Cs_2_AgFeCl_6_ shows high thermal stability up to 536 °C and high crystal stability that can be kept under normal ambient conditions for more than 63 days.^25^ Cs_2_NaFeCl_6_ also exhibits high thermal stability and reversible thermochromic behavior over a wide temperature range from 80 to 500 K.^26^ Hole conductivity can be modified via the weight contribution of Na/Ag orbital states in the Cs_2_(Ag:Na)FeCl_6_ alloys.^23^ Most recently, magnetometry measurements of Cs_2_NaFeCl_6_ and Cs_2_AgFeCl_6_ reveal AFM order with a Néel temperature (*T*_N_) at about 2.71 and 17.9 K, respectively.^27^ Yet, our understanding of AFM ordering in halide double perovskites remains at an early stage, and associated magnetic properties remain largely unexplored.

This calls for an in-depth study of the origin of the magnetic response of the stable Cs_2_(Ag:Na)FeCl_6_ alloys. The physics of a superexchange interaction pathway is interesting as it holds the key to modifying exchange interaction strength in the three-dimensional MHP network. In addition, the inherent B^III^-site order of the double perovskites crystal structure (with a nonmagnetic B^I^ site) results in a magnetic frustration due to a corner-sharing of the magnetic ions,^28,29^ making metal-halide double perovskites attractive as a new playground in a search for a novel quantum spin liquid phase.^30,31^ Combined with the flexibility in the choices of the nonmagnetic element at the B^I^ site, using multiple ions at the B^I^ site shall offer a degree of freedom to modify the superexchange pathway, which is beneficial for tuning the magnetic interaction.

## Materials and Methods

### Sample Preparation

Single crystals of the Cs_2_AgFeCl_6_, Cs_2_NaFeCl_6_, and Cs_2_Ag_0.6_Na_0.4_FeCl_6_ alloys were produced through a hydrothermal method. The starting materials, consisting of CsCl, AgCl, NaCl, FeCl_3_, and HCl, were dissolved in HCl and then transferred to a Teflon-lined autoclave. The sealed autoclave was then placed in an oven and heated to 180 °C for 12 h. Finally, it was cooled down to room temperature gradually at a rate of 1 °C per hour.

### X-ray Diffraction Experiment

The X-ray diffractometry (XRD) was carried out using an X’Pert PRO diffractometer from PANalytical, with a copper Cu Kα (λ = 1.5406 Å) source operated at 45 kV and 40 mA. The Bragg–Brentano HD mirror was used as a primary optics with a 0.5° divergence slit and a 0.020 mm nickel filter. The powder 2θ–ω measurement was set from 3° to 60° with a 0.017° step size. The XRD measurements were analyzed using Highscore software (PANalytical), which was used to extract the average background from the signal and fit the peaks.

### Electron Paramagnetic Resonance Spectroscopy

Q-band EPR was performed by using a Bruker ELEXSYS E500 spectrometer operating at about 34 GHz. The EPR signal was recorded in the dark. The crystal powder was prepared by grounding a few single crystal samples. The powders were sealed in an evacuated quartz tube and placed in the He-cryostat. The measurement temperature was in the range 3–300 K.

### Antiferromagnetic Resonance by Far-Infrared Absorption Spectroscopy

The far-infrared absorption spectroscopy was performed on a Bruker IFS113 V FT spectrometer with a mercury arc lamp source. A 50 μm beam splitter was used to measure spectra in the range from 12 to 40 cm^–1^ (360–1200 GHz). The far-infrared radiation was guided through a crystal powder sample with an aperture of 8 mm in diameter. The samples were placed in a He bath cryostat with exchange He gas at a base temperature of 1.3 K. An external magnetic field was produced by a Florida-Bitter magnet with the maximum field of 33 T available at the High Field Magnet Laboratory (HFML, Nijmegen). A silicon bolometer placed next to the sample was employed to detect the transmission signal, which was then Fourier transformed and subtracted from the zero-field signal.

## Results and Discussion

### Structural Analysis

**Figure 1:**
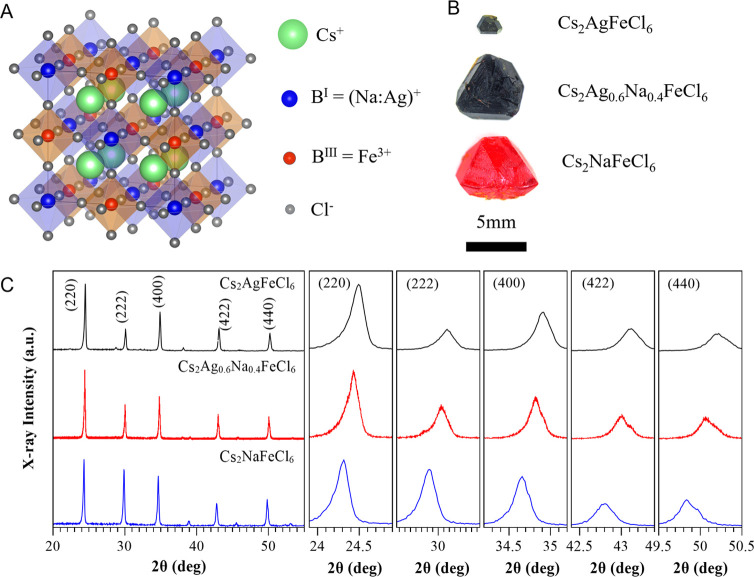
Structural properties of Cs_2_(Ag:Na)FeCl_6_ samples. (A) Cubic double perovskite crystal structure. (B) Photograph images of Cs_2_AgFeCl_6_, Cs_2_Ag_0.6_Na_0.4_FeCl_6_, and Cs_2_NaFeCl_6_ single crystals. (C) XRD patterns of Cs_2_AgFeCl_6_, Cs_2_Ag_0.6_Na_0.4_FeCl_6_, and Cs_2_NaFeCl_6_ single crystal powders with the zoom-in of the XRD peaks associated with the (220), (222), (400), (422), and (440) cubic crystallographic planes.

Figure 1 presents the main results of the structural characterization performed at room temperature. The single crystals of Cs_2_AgFeCl_6_, Cs_2_NaFeCl_6_, and Cs_2_Ag_0.6_Na_0.4_FeCl_6_ were ground into a powder for X-ray diffraction (XRD) experiments. The crystal structure of all three samples is confirmed to inherit the overall cubic double perovskite structure of symmetry *Fm*3̅*m*, as schematically shown in Figure 1A. Figure 1B shows typical photographs of our single crystals as large as 1–5 mm^2^ obtained via hydrothermal growth. The normal surface of the three samples is the (111) plane. The crystal color changes from orange to black as the atomic percentage of Ag increases, associated with bandgap reduction. The XRD patterns are plotted in Figure 1C. The cubic lattice constants for Cs_2_AgFeCl_6_ and Cs_2_NaFeCl_6_ are determined to be 1.027 ± 0.001 and 1.034 ± 0.002 nm, respectively, in agreement with a previous report.^23^ For the Cs_2_Ag_0.6_Na_0.4_FeCl_6_ crystal, the XRD peaks lie in between those of Cs_2_AgFeCl_6_ and Cs_2_NaFeCl_6_, resulting in a cubic lattice constant of 1.030 ± 0.002 nm for Cs_2_Ag_0.6_Na_0.4_FeCl_6_, exhibiting linear scaling according to the Ag:Na ratio.

### Long-Range Antiferromagnetic Order

**Figure 2:**
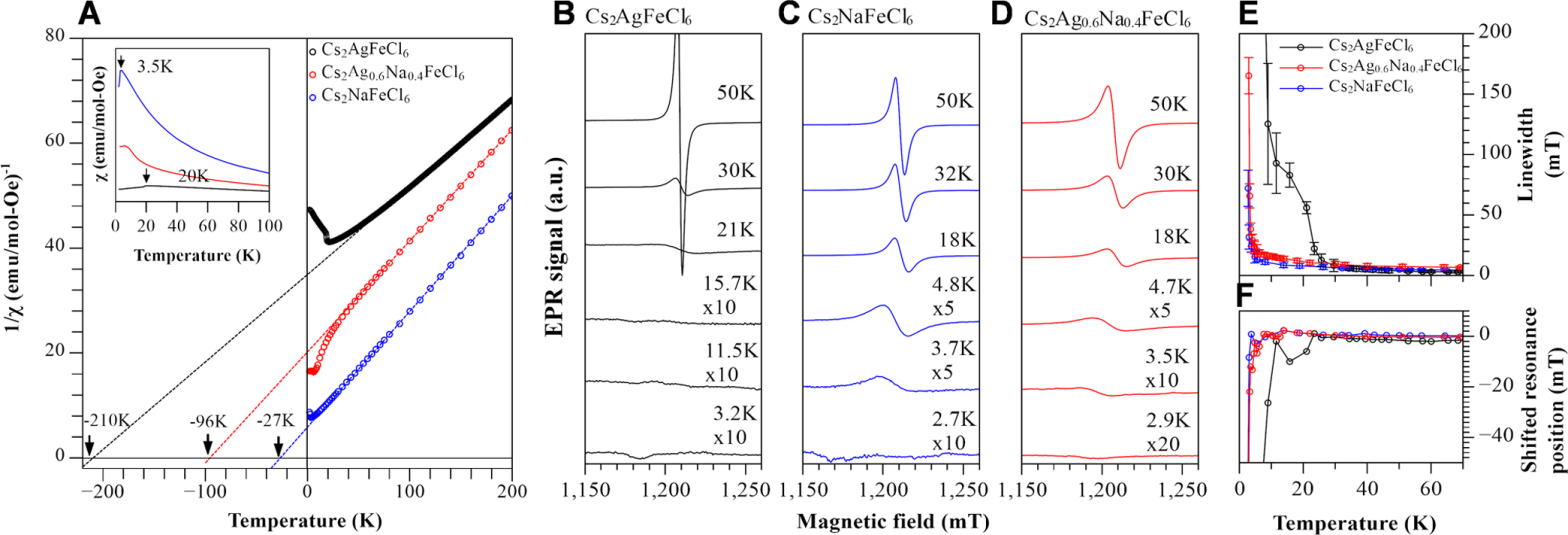
Magnetic properties of Cs_2_(Ag:Na)FeCl_6_ samples. (A) Temperature-dependent inverse magnetic susceptibility of Cs_2_(Ag:Na)FeCl_6_. (B–D) Temperature-dependent Q-band (34 GHz) EPR spectra from Cs_2_AgFeCl_6_, Cs_2_NaFeCl_6_, and Cs_2_Ag_0.6_Na_0.4_FeCl_6_. (D,E) Temperature dependence of EPR line width and the shifts of the EPR field position with respect to its position at 100 K.

Temperature-dependent magnetic susceptibility (χ) measurements were performed by using a superconducting quantum interference device (SQUID) to examine the magnetic response in the Cs_2_(Ag:Na)FeCl_6_ powder samples (shown in Figure 2A). The magnetic susceptibility curves of Cs_2_AgFeCl_6_ and Cs_2_NaFeCl_6_ first rise with decreasing temperature, followed by a decrease at further lower temperatures (inset of Figure 2A), indicating a paramagnetic-to-antiferromagnetic phase transition. The Neel temperature *T*_N_ was found to be 20 K in Cs_2_AgFeCl_6_ and 3.5 K in Cs_2_NaFeCl_6_, which are consistent with the previous report.^8^ For the Cs_2_Ag_0.6_Na_0.4_FeCl_6_ alloy, the temperature-dependent χ poses a somewhat different behavior, with only a slight drop of χ as the temperature approaches below 8 K. To exemplify this behavior, we plot 1/χ for all samples as a function of temperature and fit this dependence using the Curie–Weiss law (the main panel in Figure 2A). The effective magnetic moment (μ_eff_) was deduced from the slope of these curves, resulting in a μ_eff_ value in the range from 6.0 to 6.9 for all samples, correlating well with the Fe^3+^ ion in the high-spin *S* = 5/2 configuration in the halide octahedral environment.^27^ The fitting yields a negative Curie–Weiss temperature (θ_CW_) of −210 K (Cs_2_AgFeCl_6_), −96 K (Cs_2_Ag_0.6_Na_0.4_FeCl_6_), and −27 K (Cs_2_NaFeCl_6_), respectively, indicating a predominant AFM interaction that can be tuned via alloying on nonmagnetic B^I^ sites.

It can be clearly seen from Figure 2A that a 1/χ anomaly of Cs_2_Ag_0.6_Na_0.4_FeCl_6_ appears below 30 K, without a clear turning point to an AFM transition. This can be attributed to the alloying effect causing a local disorder and, hence, a broadening of the phase transition temperature. This renders it difficult to conclude that the long-range AFM coupling is eventually formed in the alloy case.

To gain microscopic information about the paramagnetic species and their transformation during magnetic phase transitions, we conducted in-depth electron-paramagnetic-resonance (EPR) studies in parallel to the SQUID. The temperature-dependent EPR spectra from Cs_2_AgFeCl_6_, Cs_2_NaFeCl_6_, and Cs_2_Ag_0.6_Na_0.4_FeCl_6_ are plotted in Figure 2B–D. In the paramagnetic region (*T* > 50 K, as justified by SQUID), EPR signals in all three crystals exhibit a single resonance line corresponding to spin resonance of an effective spin-1/2 paramagnetic center with *g* = 2.01. The EPR line width is rather broad (>1 mT) without a resolvable fine or hyperfine structure. It is noteworthy, however, that the EPR line width of Cs_2_Ag_0.6_Na_0.4_FeCl_6_ is broader and is twice as large as that of both Cs_2_AgFeCl_6_ and Cs_2_NaFeCl_6._ To identify the origin of the EPR signal, we synthesized a series of Fe-doped Cs_2_(Ag:Na)InCl_6_ (Fe < 1%) and performed temperature-dependent X-band EPR studies as shown in Figure S1A–C. Here, large separations between Fe^3+^ ions resulting from a low Fe concentration and the In^3+^ acting as a nonmagnetic spacer reduce the exchange interaction between Fe^3+^, thereby reducing the broadening of the EPR signal due to the spin exchange interaction. For Fe-doped Cs_2_AgInCl_6_ and Cs_2_NaInCl_6_, a single narrow resonance line is observed with no fine-structure splitting, whereas EPR spectra from Fe-doped Cs_2_Ag_0.6_Na_0.4_InCl_6_ exhibit multiple resonance lines. A spin-Hamiltonian analysis of the EPR spectra from the Fe-doped Cs_2_Ag_0.6_Na_0.4_InCl_6_ suggests an *S* = 5/2 spin state with a local symmetry lower than the cubic of the bulk (Figure S1D,E). The most likely origin of the EPR signal is high-spin Fe^3+^ residing on a B^III^ site, with a reduced local symmetry and a likely distorted Fe^3+^-Cl_6_ octahedron due to random and asymmetric placements of Ag and Na on its nearest neighbor B^I^ sites. The resulting low-symmetry crystal field leads to a fine-structure splitting of the *S* = 5/2 spin state, and thus the observed multiline pattern of the EPR signal. On the other hand, as such random and asymmetric placements of different B^I^ ions is absent in Fe-doped Cs_2_AgInCl_6_ and Cs_2_NaInCl_6_, the EPR signals in these two crystals are associated with the same high-spin *S* = 5/2 Fe^3+^ but in the cubic symmetry without the zero-field splitting of the spin sublevels when all EPR lines are merged into the same field.^32^ Therefore, we can conclude that the presence of an unresolved fine structure caused by a local crystal field of various symmetry and strength should significantly contribute to the observed further broadening of the EPR line width in Cs_2_Ag_0.6_Na_0.4_FeCl_6_. The fact that the fine structure is unresolved in Cs_2_Ag_0.6_Na_0.4_FeCl_6_ whereas it is clearly resolved in Cs_2_(Ag:Na)InCl_6_ with a dilute Fe^3+^ concentration suggests that the spin superexchange interaction should also play a role in the EPR line broadening of the former. Together with the deduced μ_eff_ values from SQUID, we conclude that the EPR signal in Cs_2_AgFeCl_6_ and Cs_2_NaFeCl_6_ at *T* > 50 K originates from the *S* = 5/2 Fe^3+^ paramagnetic center of cubic symmetry, whereas the broader EPR signal from the Cs_2_Ag_0.6_Na_0.4_FeCl_6_ alloy arises from the *S* = 5/2 Fe^3+^ with a reduced local symmetry.

As shown in Figure 2B–D, the EPR line width in all three samples becomes broadened as temperature decreases to a critical value, after which the EPR signals eventually disappear. This is accompanied by a shift of the resonance position toward a low magnetic field. By fitting the measured signals by a derivative Lorentzian line, the line width and the shift of the resonance position can be deduced and are plotted in Figure 2E,F as a function of temperature. For Cs_2_AgFeCl_6_ and Cs_2_NaFeCl_6_, both the line width broadening and the peak shift directly correlate with the magnetic susceptibility data from the SQUID. Both exhibit a sudden change at the Neel temperature (20 and 3.5 K, respectively). This EPR behavior near the AFM transition is well described in the literature.^33−35^ The broadening is associated with increased long-range magnetic order. The shift in the resonance position is attributed to fractional net magnetization within the magnetic sublattice that acts on the paramagnetic states before forming a complete AFM order.

The EPR results confirm that the long-range AFM order in the Cs_2_Ag_0.6_Na_0.4_FeCl_6_ alloy is eventually formed but at a much lower temperature than what is anticipated from its Curie–Weiss temperature. Instead of lying nearly midway between those of Cs_2_AgFeCl_6_ and Cs_2_NaFeCl_6_, the Neel temperature is seen to be much closer to that of Cs_2_NaFeCl_6_. Due to a large experimental error bar, unfortunately, the exact value of *T*_N_ cannot be determined but can be estimated to lie within the range of 3.5–8 K, which is consistent with our SQUID data. This signifies the benefit of the ESR technique in studying a long-range magnetic coupling, complementary to the SQUID technique. The magnetic parameters deduced from the SQUID and EPR analyses are summarized in Table 1. We determine the frustration factor *f* = |θ_CW_|/*T*_N_ as a degree of deviation between the measured AFM interaction strength (∝|θ_CW_|) and the measured *T*_N_. Both Cs_2_AgFeCl_6_ and Cs_2_NaFeCl_6_ exhibit a quite similar value of *f* ∼ 10. On the other hand, we speculate an enhanced magnetic frustration with a magnitude as large as 12–27 is observed for Cs_2_Ag_0.6_Na_0.4_FeCl_6_. This observation might indicate a possible link between alloy disorder and magnetic destabilization.

**Table 1:** Magnetic Parameters of Cs_2_(Ag:Na)FeCl_6_^a^

**Parameter**	**Cs**_**2**_**AgFeCl**_**6**_	**Cs**_**2**_**NaFeCl**_**6**_	**Cs**_**2**_**Ag**_**0.6**_**Na**_**0.4**_**FeCl**_**6**_
**Curie–Weiss temperature (*****θ***_**CW**_**)**	–210 K	–27 K	–96 K
**Néel temperatures (*****T***_**N**_**)**	17–20 K	2.7–3.5 K	3.5–8 K
**Frustration factor (*****f*****)**	10.5–12.4	7.7–10	12–27
**Effective magnetic moment (*****μ***_**eff**_**)**	6.935 μ_B_	6.021 μ_B_	6.150 μ_B_

a All parameters were deduced from a combination of both SQUID and EPR results.

**Figure 3:**
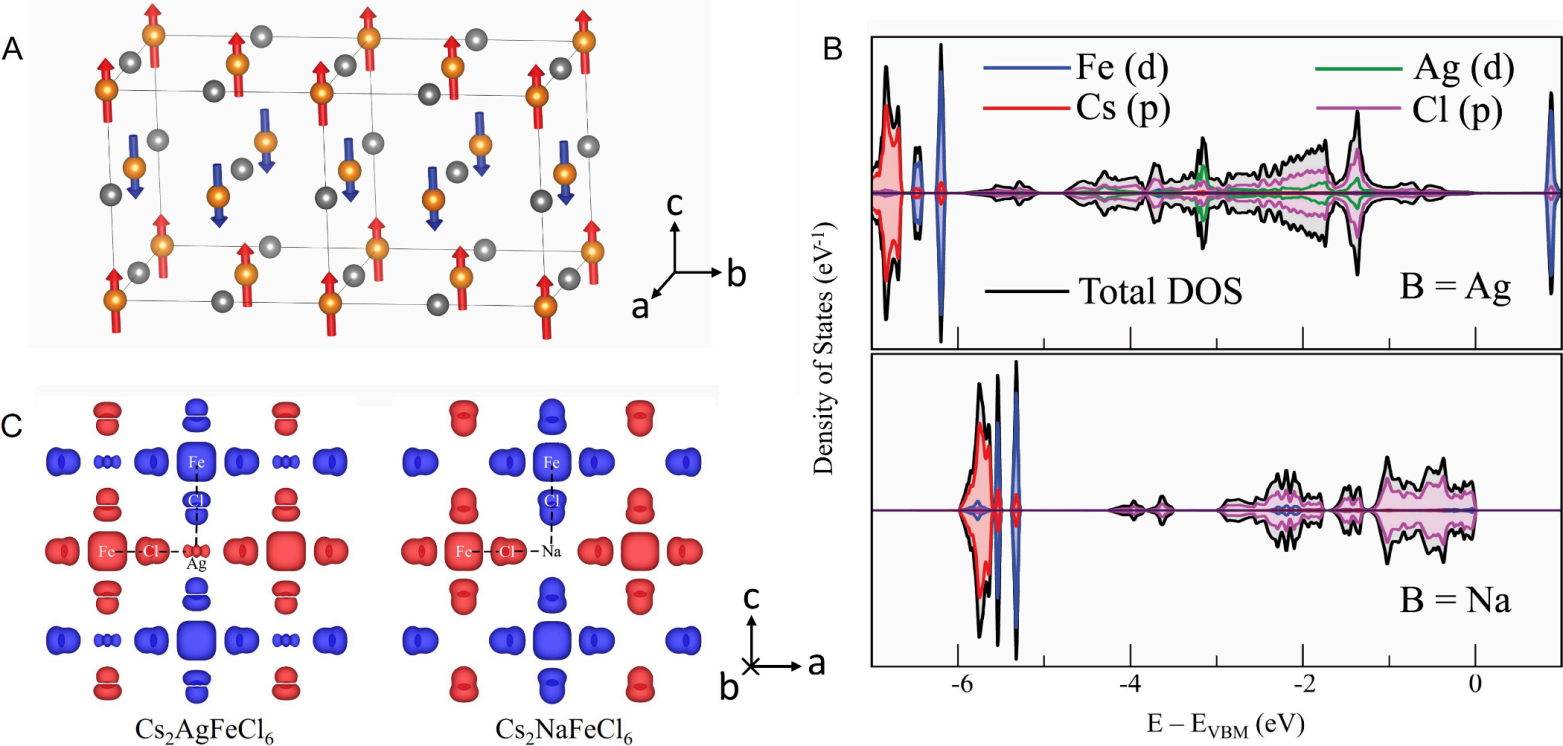
Simulated antiferromagnetic properties of Cs_2_(Ag:Na)FeCl_6_ samples at 0 K. (A) Illustrated AFM type-1 magnetic configuration for A_2_B^I^B^III^X_6_ (B^I^ = Ag, Na) structure. The arrows indicate the magnetization direction of Fe ions. (B) Partial density of states for Cs_2_AgFeCl_6_ and Cs_2_NaFeCl_6_. The valence band maxima are set to zero. (C) Spin up (red) and down (blue) density, along the *ac*-plane cross-section, for Cs_2_AgFeCl_6_ and Cs_2_NaFeCl_6_ with an isosurface value of 0.002 e^–^/Å^3^. Fe–Cl–Ag:Na–Cl–Fe pathways are illustrated with dashed lines. Notice the presence and absence of spin density on Ag and Na ions, respectively.

To better understand the role of the B^I^ monovalent species in facilitating the magnetic order, we performed electronic structure calculations at the Density Functional Theory (DFT) level using a classic Heisenberg Hamiltonian. The details of these calculations can be found in the Supporting Information. For Cs_2_AgFeCl_6_, the magnetic exchange interactions between an Fe atom and its *i*^*th*^ nearest neighbor of the Fe atoms, *J*_*i*_, are calculated to be −3.01 (*J*_1_), −0.42 (*J*_2_), −0.52 (*J*_3_), and −0.24 meV (*J*_4_). For Cs_2_NaFeCl_6_ we obtain −0.38 (*J*_1_), −0.015 (*J*_2_), −0.064 (*J*_3_), and −0.006 meV (*J*_4_). We calculate the anisotropy constants (*K*_*x*_, *K*_*y*_, *K*_*z*_) to be (0.0000, 0.0000, 0.001) meV for B^I^ = Ag, and expectedly, those for B^I^ = Na are much smaller at (0.0000, 0.0000, 0.0003) meV. Subsequently, we perform Classical Monte Carlo simulations to determine the Neel temperatures (*T*_N_) and the Curie–Weiss parameters (θ_CW_). The resulting values for Cs_2_AgFeCl_6_ are 37 K (*T*_N_) and −292 K (θ_CW_), while for Cs_2_NaFeCl_6_ we find 8 K (*T*_N_) and −50 K (θ_CW_). These values are quite close to the experimental results, indicating that the Heisenberg Hamiltonian models the magnetic interactions in the system reasonably well. Our analysis of the exchange interactions in Cs_2_AgFeCl_6_ and Cs_2_NaFeCl_6_ reveals that the interactions beyond the nearest neighbor Fe atoms are relatively weak (*J*_1_ > *J*_2_, *J*_3_, *J*_4_). This shows that the dominant superexchange interaction occurs between the Fe nearest neighbors (*J*_1_) through the Fe–Cl–(Ag:Na)–Cl–Fe pathway (schematically shown in Figure S2).^36^ This is expected given the relatively large interatomic distance between the magnetic species in these materials. Electronic structure calculations predict that the spin configuration in Cs_2_AgFeCl_6_ and Cs_2_NaFeCl_6_ is type-1 AFM, where Fe magnetic moment vectors alternate along the *c*-crystallographic direction but have the same orientation in the *ab*-plane,^37^ illustrated as red(blue) arrows in Figure 3A.

However, it is interesting that the value of *J*_1_ is an order of magnitude larger in Cs_2_AgFeCl_6_ than in Cs_2_NaFeCl_6_, pointing to possible differences in the exchange mechanisms between these two compounds. Another notable difference between these two materials is the strong p–d hybridization between Cl (p) and Ag (d) orbitals, judging from the similar DOS characters of the two orbital states near the valent band maxima. This occurs only when Ag occupies the B^I^ site, as illustrated in Figure 3B. To quantify the strength of hybridization, we calculate the Crystal Orbital Hamilton Population (COHP) for each pair of atoms.^38^ The COHP values integrated up to the Fermi level (ICOHP) can then be used to compare the strength of the hybridization between the different ions. As summarized in Table S1, the hybridization between mediating atoms Ag and Cl is stronger than that between Na and Cl. Naturally, the exchange mechanism might be influenced by the type of interaction between the mediating the atoms. To further investigate the role of hybridization in facilitating magnetic order, we plot the spin densities for both compounds in the energetically favorable AFM type-1 configuration (Figure 3C). We note that all “Cl” atoms in the [FeCl_6_]^3–^ octahedra assume the same spin as the central “Fe” atom. In the case of Cs_2_AgFeCl_6_, a nonzero spin density on “Ag” is observed in addition to the “Cl” atoms, meaning that the Ag (d^10^s^0^) ions are also spin-polarized owing to p–d hybridization. In contrast, for the same isosurface value in Cs_2_NaFeCl_6_, no spin density is observed on “Na” (p^6^s^0^) ions, which is attributed to the lack of covalent Na(s) and Cl(p) interactions in the system, as observed clearly in the density of states. Thus, the hybridized Ag(d)–Cl(p) orbitals in Cs_2_AgFeCl_6_ are more effective at mediating exchange interactions in the double-perovskite structure compared to Cl(p) orbitals in Cs_2_NaFeCl_6_. This emphasizes the importance of the choice of monovalent species occupying the B^I^ site in the double perovskite A_2_B^I^B^III^X_6_ structure, as it may prove to be central to the design of magnetic halide double perovskites. Furthermore, we calculate orbital decomposed exchange interactions and deduce that only the doubly degenerate e_g_ = (d_*x*^2^ – *y*^2^_, d_*z*^2^_) set of orbitals participate in the exchange interactions between any two Fe atoms. With respect to the AFM-1 structure, if the two Fe spins assume the same direction, most exchange interactions mediated by the B^I^ cation are effectively between the d_*x*^2^–*y*^2^_ orbitals on both atoms, while if the two Fe atoms assume opposite spins, most B^I^-mediated exchange interactions are between d_*x*^2^–*y*^2^_ orbitals and d_*z*^2^_ orbitals on the first and second Fe atom, respectively. While these two pathways dominate in both B^I^ = Ag, Na structures, the strength of these interactions is severely diminished when the monovalent cation is Na^+^ due to the reasons described above.

For Cs_2_Ag_0.6_Na_0.4_FeCl_6,_ we model the density of state and account for the alloy distributions via a special quasi-random structure involving a supercell of 160 atoms. It is noteworthy to point out the lesser number of Ag(d) states near the valence band maxima with respect to Cl(p) states as compared to the pristine Cs_2_AgFeCl_6._ This points out the lesser degree of the exchange interaction mediated by p–d hybridization. However, we note that both θ_CW_ and *T*_N_ for Cs_2_Ag_0.6_Na_0.4_FeCl_6_ cannot be accurately predicted in our model. The complication arises due to the unknown local structure of the alloys. To explain the possible enhanced magnetic fluctuation, further studies associated with the local magnetic structure are needed.

It is also worth mentioning that below the phase transition of Cs_2_AgFeCl_6_, the DFT calculation predicts a magneto-structural transition, where the spin-polarized Ag (d^10^s^0^) seems responsible for the tetragonal distortion along the *c*-crystallographic direction with a predicted tetragonal distortion ratio of about 0.98. This results in an easy axis AFM-1 with weak magnetic anisotropy in Cs_2_AgFeCl_6,_ whereas Cs_2_NaFeCl_6_ and Cs_2_Ag_0.6_Na_0.4_FeCl_6_ restrain a cubic character in the AFM-1 configuration. To further investigate into this, we perform a supplemental antiferromagnetic resonance (AFMR) study through, i.e., far-infrared absorption in a high magnetic field; see Figure S3. Unfortunately, due to our instrumental limitation (360–750 GHz far-infrared radiation), we can only probe AFMR of the magnon in the range of magnetic field *B* > 13 T. This prevented us from accurately determining the magnetic anisotropy parameter of Cs_2_AgFeCl_6_. Nevertheless, the line width analysis of the AFMR suggests that the magnetic anisotropy does exist in Cs_2_AgFeCl_6_, with the anisotropy parameter close to the range predicted by the DFT calculations.

In terms of possible applications of the tunable Curie–Weiss parameters as studied in this work, we foresee thermal switch using magnetic materials.^39,40^ Here, a higher operational temperature close to and preferably at room temperature would be essential. This requires further in-depth investigations into optimal choices of material design to promote exchange interactions, e.g., through itinerated electrons via doping and/or light-induced carriers.

## Conclusion

We have studied the magnetic properties of halide double perovskites Cs_2_(Ag:Na)FeCl_6_ by a combination of experimental studies (including SQUID and magnetic resonance) and DFT calculations. All three single crystals of Cs_2_AgFeCl_6_, Cs_2_Ag_0.6_Na_0.4_FeCl_6_, and Cs_2_NaFeCl_6_ exhibit AFM ordering with Neel temperatures of 20, 3.5–8, and 3.5 K, respectively. A large deviation from their Curie–Weiss temperatures of −210 K (Cs_2_AgFeCl_6_), −96 K (Cs_2_Ag_0.6_Na_0.4_FeCl_6_), and −27 K (Cs_2_NaFeCl_6_) indicates an inherent magnetic frustration. The orbital character of Ag (4d^10^5s^0^) and Na (2p^6^3s^0^) is shown to determine the superexchange interaction pathway, either via the route Fe–Cl–Ag–Cl–Fe or bypass in Na (2p^6^3s^0^) due to a lack of Cl–Na hybridization, leading to the ability to tune θ_CW_ by (Ag:Na) alloying. A possible increase of the magnetic frustration factor observed in the Ag:Na alloy can be attributed to the disorder-induced local octahedral distortion destabilizing the long-range magnetic order. In this work, we have provided a general understanding of the importance of the nonmagnetic B^I^ site in facilitating superexchange interactions and its contribution to long-range magnetic ordering, magnetic anisotropy, and magneto-structural transition, which could serve as a guideline in our search for and optimization of magnetic perovskites for future applications in spintronics and quantum information technology.

## Data Availability

All data are available upon a reasonable request to the corresponding author.

## Supporting Information Available

Method description for the density functional theory calculations, the figures for (1) EPR spectra of 1% Fe-doped Cs_2_AgInCl_6_, Cs_2_NaInCl_6_, and Cs_2_Ag_0.6_Na_0.4_InCl_6_, (2) schematic for the superexchange interaction pathway of J_1_ and J_2_ inside a Cs_2_(Ag:Na)FeCl_6_ crystal, (3) AFMR of the Cs_2_(Ag:Na)FeCl_6_ samples, and (4) density of states for Cs_2_(Ag_0.6_Na_0.4_)FeCl_6_ alloy modeled via a special quasi-random structure, table of hybridization data (PDF)
