# Supplementary material for: Understanding Antiferromagnetic Coupling in Lead-Free Halide Double Perovskite Semiconductors
Source: J Phys Chem C Nanomater Interfaces. 2024 Mar 14;128(12):5313–20. doi: 10.1021/acs.jpcc.3c08129 (PMC10982993; doi:10.1021/acs.jpcc.3c08129)
Supplement: Supplementary file 1 — jp3c08129_si_001.pdf [file jp3c08129_si_001.pdf]

Supplementary Materials

Understanding Antiferromagnetic Coupling in Lead-free Halide Double Perovskites Semiconductors

Authors: Kunpot Mopoung[1], Weihua Ning[2], Muyi Zhang[1], Fuxiang Ji[1], Kingshuk Mukhuti[3], Hans Engelkamp[3], Peter M. Christianen[3], Utkarsh Singh[1], Johan Klarbring[1], Sergei I. Simak[1,4], Igor A. Abrikosov[1], Feng Gao[1], Irina A. Buyanova[1], Weimin M. Chen[1]*, Yuttapoom Puttisong[1]*

[1]Deperment of Physics, Chemistry, and Biology (IFM), Linköping University, Linköping 583 30, Sweden.
[2]Institute of Functional Nano & Soft Materials (FUNSOM), Soochow University, Suzhou 215123, P.R. China.
[3]High Field Magnet Laboratory (HFML - EMFL), Radboud University, Nijmegen 6525 ED, The Netherlands.
[4] Department of Physics and Astronomy, Uppsala University, Uppsala SE-75120, Sweden.
*e-mail-address weimin.chen@liu.se, yuttapoom.puttisong@liu.se

Supporting Information

Density functional theory (DFT) calculations

The DFT calculations were performed in the Vienna ab initio simulation package (VASP) [1–3] using the projector augmented - wave (PAW) [4] method with a plane wave cut-off of 450 eV for all calculations. We performed full structural relaxation with a convergence criterion of 10^{-8} eV for total energy in self-consistent field cycles and < 5 meV/Å for atomic forces. A k-point density of 0.15 $Å^{-1}$ for structural relaxations and 0.10 $Å^{-1}$ for accurate total energy calculations was used throughout.

The PBEsol DFT functional with Hubbard U correction was used for geometry optimization as well as the calculation of density of states [5–7]. We chose the value of U = 3 eV, which has recently been shown to be comparable to hybrid functionals when reproducing the electronic and magnetic properties of these systems [8]. The calculations of the first four nearest-neighbour interactions were performed in an 80 - atom supercell.

We used the TB2J [9] package to calculate orbital decomposed exchange interactions which is based on Green's function method of calculating exchange interaction proposed by Liechtenstein et al [10].

The Monte-Carlo calculations were performed with the UppASD code using the exchange interactions obtained via Density functional theory calculations [11].

In general, magnetic Hamiltonian can be assumed as

$$H_{mag} = -\sum_{\langle i,j\rangle} J_{ij} S_i \cdot S_j + \sum_{\langle i,j\rangle} D_{ij} S_i \times S_j + \sum_{\langle i,j\rangle_\gamma} K_\gamma S_i^\gamma S_j^\gamma + \sum_{\langle i\rangle} \sum_{x,y,z} K_x (S_i^x)^2$$

Here, owing to weak relativistic effects, the non-linear Dzyaloshinskii – Moriya interactions (D_{ij}) and Kitaev interactions (K_γ) are expected not to contribute significantly compared to the dominant Heisenberg exchange interactions term J_{ij} and anisotropy constants (K_x, K_y, K_z). S_i and S_j represent the magnitudes of spins of atoms i and j.

Therefore, the magnetic Hamiltonian reduces to a simple Heisenberg Hamiltonian

$$H_{mag} = -\sum_{\langle i,j\rangle} J_{ij} S_i \cdot S_j + \sum_{\langle i\rangle} \sum_{x,y,z} K_x (S_i^x)^2$$

We then extracted the exchange interactions between any two sites i and j given by

$$J_{ij} = \frac{(E_{\uparrow\downarrow} + E_{\uparrow\downarrow} - E_{\uparrow\uparrow} - E_{\downarrow\downarrow})}{4}$$

Here, $E_{\uparrow\downarrow}$ refers to the energy of the system when the spins at sites i and j are aligned antiparallel to each other w.r.t. a reference configuration, which is assumed to be AFM-1 for our calculation. For simplicity, the exchange interaction between an Fe atom and its ith nearest neighbour can be represented by J_i. The anisotropy constants were calculated using the difference in calculated energy between configurations with spins aligned along different crystal directions within the same magnetic structure. Since the crystal is symmetric along x and y axes, change in direction of the magnetization vector within the x - y plane does not change the total energy, while that in y - z or x - z plane does. Thus, here we have $K_x = K_y = 0$ while $K_z = (\langle E\rangle_{easy-axis} - \langle E\rangle_{hard-axis})/2$ where E represents the total energy per atom when spins are aligned along easy [001] and hard [010] axes.

Finally, the magnetic transition temperatures were obtained by performing a temperature sweep starting from a temperature of 600 K and going down to 2 K in order to observe the magnetic phase transition. At each temperature step, the system was equilibrated through 10^5 steps before sampling the configuration space (C.S.). Since we deal with low temperatures, the Heat Bath Monte Carlo algorithm was used to effectively sample the C.S.

The pathway of the exchange interaction of the spin are shown in Fig. S2. In this work, the atomic spins are set to unit magnitude and should be scaled appropriately while making a comparison to works where this is not the case.

The 0K DFT calculations also capture the tetragonal distortion in case of AFM -1 magnetic configuration of $Cs_2AgFeCl_6$. The same is not observed for the ferromagnetic configuration on the Fe sublattice, indicating that the distortion is likely owed to presence of spin - lattice coupling, which leads to the stabilization of low temperature AFM-1 phase. The fact that this distortion and resulting anisotropy is nearly negligible in case of the stable AFM phase for $Cs_2NaFeCl_6$, also highlights the difference in the strength of magnetic exchange interactions owing to the choice of mediating monovalent cation. Details on chemical bonding were obtained from a crystal orbital Hamiltonian population (COHP)[12] analysis as implemented in the LOBSTER package[13–15].

The antiferromagnetic resonance and the magnetic anisotropy

We used an antiferromagnetic resonance (AFMR) technique to examine antiferromagnetic properties (e.g. magnon modes and magnetic anisotropy). According to the measured Curie-Weiss parameters and the DFT predictions, we expect the AFMR frequency of all samples to be beyond a standard X- to W-band (10-100 GHz) EPR spectrometer. Therefore, we resorted to high-frequency/high-magnetic field, far-infrared absorption experiments. Here, 360-750 GHz far-infrared radiation was applied to the powder samples of all crystals. The transmission was detected via a He-cooled barometer with an applied magnetic field in the range of 10-30 Tesla (the range where the free electron spin resonance condition is expected). The sample temperature was kept at 1.7K, where all three samples were in the AFM state.

The AFMR results are shown in **Figure S3A-C**. Under these conditions, the AFMR frequency in all three samples scales linearly with the magnetic field strength (see **Figure S3D**). This implies that, within this field range, the Zeeman interaction dominates over the AFM exchange interaction. The linear field dependence yields a g-factor of 2.02±0.01 in all samples, in agreement with our EPR results of the Fe^{3+} on the B^{III} site. The observed dominance of the Zeeman interaction suggests that magnon frequencies in all samples are below our instrumental limit (<360 GHz). While it is only possible to accurately determine the magnetic anisotropy by resolving the magnon modes and the critical spin-flop field, the AFMR linewidth at low resonant frequencies could be used to examine the magnetic anisotropy present in the samples. At a low AFM frequency, the contribution of the antiferromagnetic spin-exchange term emerges. In a powder system, low-frequency AFMR modes at fixed resonant fields spread over a broad range of frequency due to the AFM anisotropy, resulting in linewidth broadening. The low-frequency linewidth broadening is seen in $Cs_2AgFeCl_6$, indicating that the anisotropy term plays a role in the field range of 14-16T. This strengthens our DFT prediction for magnetic anisotropy in $Cs_2AgFeCl_6$. For both $Cs_2NaFeCl_6$ and $Cs_2Ag_{0.6}Na_{0.4}FeCl_6$, the DFT calculation predicts much less magnetic anisotropy and, therefore, no observable effect of magnetic anisotropy over the range of AFMR frequency of 400-750 GHz, in agreement with our experimental observation.

Figure S1.

The EPR spectra of 1% Fe-doped **(A)** $Cs_2AgInCl_6$, **(B)** $Cs_2NaInCl_6$ and **(C)** $Cs_2Ag_{0.6}Na_{0.4}InCl_6$ alloy at a frequency of 9.63 GHz (X-band) and the specified temperatures. The simulations in **(C)** include Fe^{+3} with tetragonal symmetry, using S = 5/2 , g = 2.02±0.01, D = 1260 ±50 MHz (the blue curve), and an unknown signal with an effective spin S = 1/2, g = 2.008±0.005 (the red curve). The predicted fan diagram of the spin sublevels of Fe^{3+} (S = 5/2), and the expected EPR transitions at the X-band frequency (the red vertical lines) in **(D)** cubic and **(E)** tetragonal symmetry, simulated along the B//[001] direction.

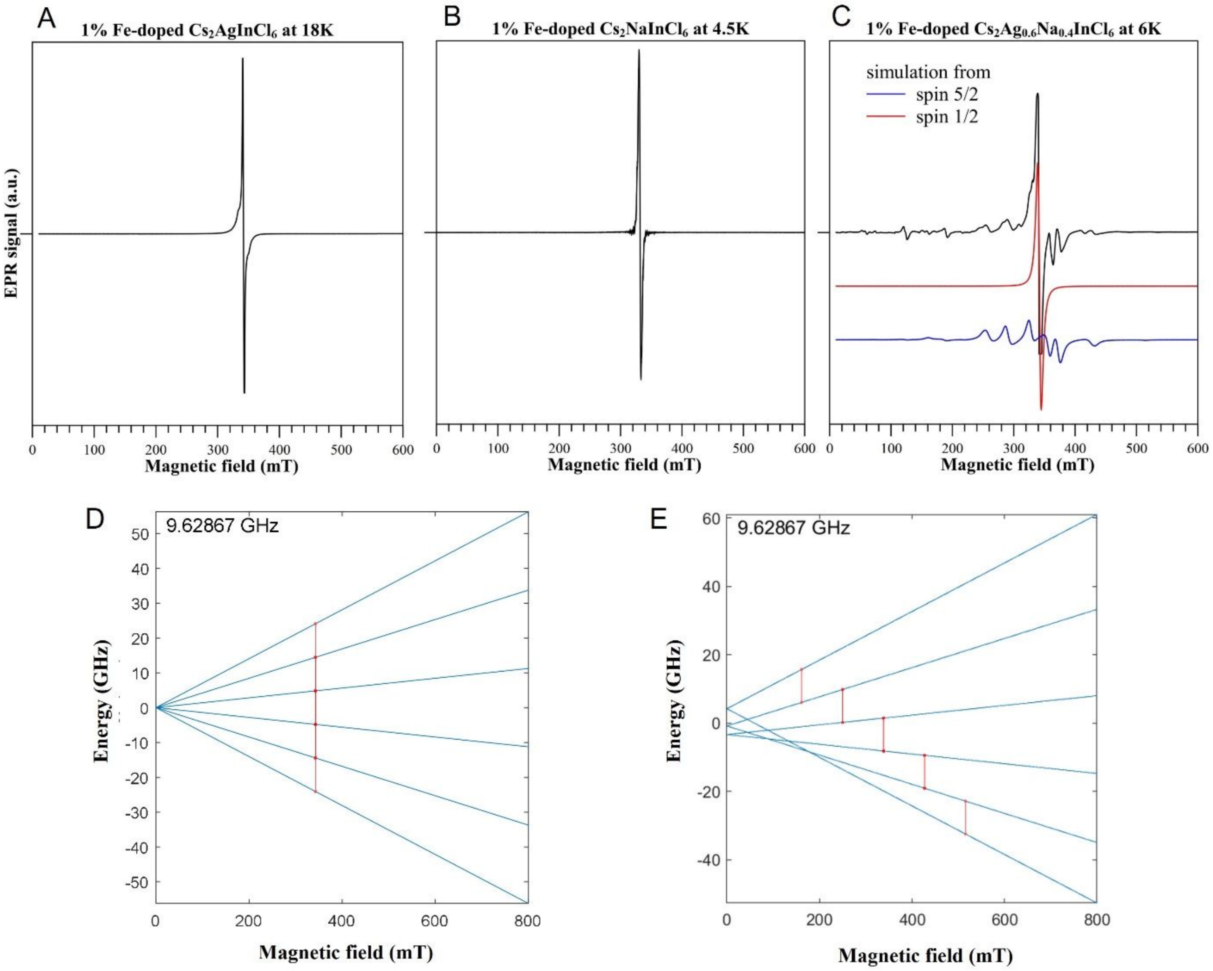

Figure S2.

The super-exchange interaction pathway of J_1 and J_2 inside a $Cs_2(Ag{:}Na)FeCl_6$ crystal.

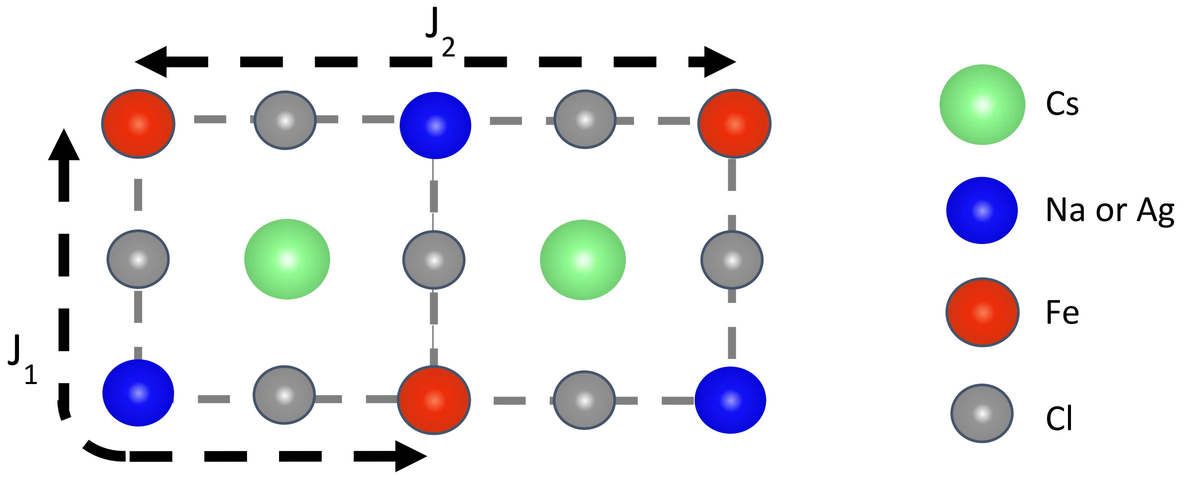

Figure S3.

Antiferromagnetic resonance (AFMR) of the $Cs_2(Ag:Na)FeCl_6$ samples at 4K. **(A-C)** AFMR line shapes of $Cs_2AgFeCl_6$, $Cs_2NaFeCl_6$, and $Cs_2Ag_{0.6}Na_{0.4}FeCl_6$, respectively. The resonant frequency at each different field point is shifted to zero for easy comparison. **(D-E)** The deduced antiferromagnetic resonance frequency and linewidth as a function of magnetic field.

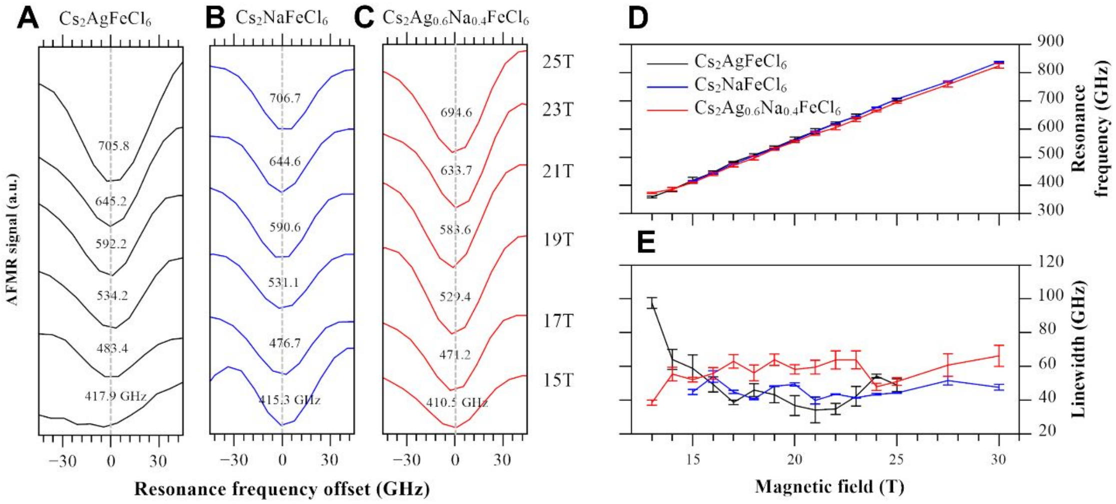

Figure S4.

The density of states for the $Cs_2(Ag_{0.6}Na_{0.4})FeCl_6$ alloy modelled via a special quasi-random structure. A supercell with a random distribution of Ag and Na atoms on the B^I site is considered. Owing to a lower concentration, lesser number of Ag(d) states near the valence band maxima with respect to Cl(p) states are observed in comparison to pristine $Cs_2AgFeCl_6$ double perovskite.

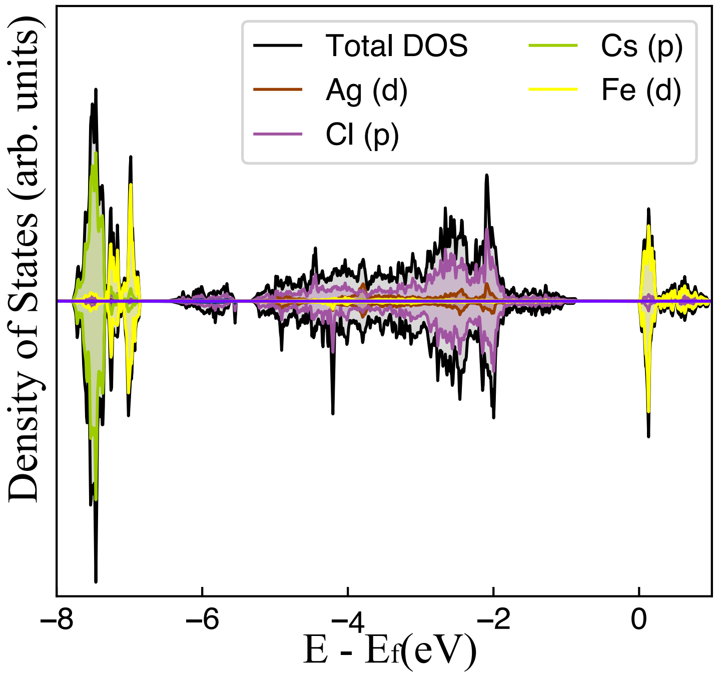

Table S1. The hybridization between Cl(p) and Ag(d) orbitals by calculating Crystal Orbital Hamilton Population.

A	B^{I}	B^{III}	X	B^{I} - B^{III}	B^{I} - X	B^{III} - X	$T_{mag.}$
Cs	Ag	Fe	Cl	0.010	0.340	0.760	20 K (this work)
Cs	Na	Fe	Cl	0.002	0.281	0.784	3.5 K (this work)
Sr	Mo	Fe	O	0.099	1.681	0.718	450 K[16]

References

1. Kresse, G. & Furthmüller, J. Efficiency of ab-initio total energy calculations for metals and semiconductors using a plane-wave basis set. *Comput. Mater. Sci.* **6**, 15–50 (1996).

2. Kresse, G. & Joubert, D. From ultrasoft pseudopotentials to the projector augmented-wave method. *Phys. Rev. B* **59**, 1758 (1999).

3. Kresse, G. & Furthmüller, J. Efficient iterative schemes for *ab initio* total-energy calculations using a plane-wave basis set. *Phys. Rev. B* **54**, 11169 (1996).

4. Blöchl, P. E. Projector augmented-wave method. *Phys. Rev. B* **50**, 17953 (1994).

5. Perdew, J. P. *et al.* Restoring the density-gradient expansion for exchange in solids and surfaces. *Phys. Rev. Lett.* **100**, 136406 (2008).

6. Liechtenstein, A. I., Anisimov, V. I. & Zaanen, J. Density-functional theory and strong interactions: Orbital ordering in Mott-Hubbard insulators. *Phys. Rev. B* **52**, R5467 (1995).

7. Dudarev, S. L., Botton, G. A., Savrasov, S. Y., Humphreys, C. J. & Sutton, A. P. Electron-energy-loss spectra and the structural stability of nickel oxide: An LSDA+U study. *Phys. Rev. B* **57**, 1505 (1998).

8. Klarbring, J., Singh, U., Simak, S. I. & Abrikosov, I. A. Electronic structure of the magnetic halide double perovskites Cs2(Ag,Na)FeCl6 from first-principles. (2022).

9. He, X., Helbig, N., Verstraete, M. J. & Bousquet, E. TB2J: A python package for computing magnetic interaction parameters. *Comput. Phys. Commun.* **264**, 107938 (2021).

10. Liechtenstein, A. I., Katsnelson, M. I., Antropov, V. P. & Gubanov, V. A. Local spin density functional approach to the theory of exchange interactions in ferromagnetic metals and alloys. *J. Magn. Magn. Mater.* **67**, 65–74 (1987).

11. Skubic, B., Hellsvik, J., Nordström, L. & Eriksson, O. A method for atomistic spin dynamics simulations: implementation and examples. *J. Phys. Condens. Matter* **20**, 315203 (2008).

12. Dronskowski, R. & Blochl, P. E. Crystal orbital Hamilton populations (COHP): energy-resolved visualization of chemical bonding in solids based on density-functional calculations. *J. Phys. Chem.* **97**, 8617–8624 (1993).

13. Deringer, V. L., Tchougréeff, A. L. & Dronskowski, R. Crystal orbital Hamilton population (COHP) analysis as projected from plane-wave basis sets. *J. Phys. Chem. A* **115**, 5461–5466 (2011).

14. Maintz, S., Deringer, V. L., Tchougréeff, A. L. & Dronskowski, R. Analytic projection from plane-

wave and PAW wavefunctions and application to chemical-bonding analysis in solids. *J. Comput. Chem.* **34**, 2557–2567 (2013).

15. Nelson, R. *et al.* LOBSTER: Local orbital projections, atomic charges, and chemical-bonding analysis from projector-augmented-wave-based density-functional theory. *J. Comput. Chem.* **41**, 1931–1940 (2020).

16. Kobayashi, K., Kimura, T., Sawada, H., Terakura, K. & Tokura, Y. Room-temperature magnetoresistance in an oxide material with an ordered double-perovskite structure. *Nature* **395**, 677–680 (1998).
